# The Relationship between Zinc Levels and Autism: A Systematic Review and Meta-analysis

**Published:** 2016

**Authors:** Nasim BABAKNEJAD, Fatemeh SAYEHMIRI, Kourosh SAYEHMIRI, Ashraf MOHAMADKHANI, Somaye BAHRAMI

**Affiliations:** 1 Research Committee, School of Medicine, Ilam University of Medical Sciences, Ilam, Iran.; 2 Department of Social Medicine, School of Medicine, Ilam University of Medical Sciences, Ilam, Iran.; 3 Department of Biostatistics, Research Center for Prevention of psychosocial impairment, University of Ilam, Ilam, Iran.; 4 Liver and Pancreatobiliary Diseases Research Center, Digestive Diseases Research Institute, Tehran University of Medical Sciences, Tehran, Iran.

**Keywords:** Zinc concentration, Autism Spectrum Disorders, Meta-analysis

## Abstract

**Objective:**

Autism is a complex behaviorally defined disorder.There is a relationship between zinc (Zn) levels in autistic patients and development of pathogenesis, but the conclusion is not permanent.

**Materials & Methods:**

The present study conducted to estimate this probability using meta-analysis method. In this study, Fixed Effect Model, twelve articles published from 1978 to 2012 were selected by searching Google scholar, PubMed, ISI Web of Science, and Scopus and information were analyzed. I² statistics were calculated to examine heterogeneity. The information was analyzed using R and STATA Ver. 12.2.

**Results:**

There was no significant statistical difference between hair, nail, and teeth Zn levels between controls and autistic patients: -0.471 [95% confidence interval (95% CI): -1.172 to 0.231]. There was significant statistical difference between plasma Zn concentration and autistic patients besides healthy controls: -0.253 (95% CI: 0.498 to -0.007). Using a Random Effect Model, the overall Integration of data from the two groups was -0.414 (95% CI: -0.878 to -0.051).

**Conclusion:**

Based on sensitivity analysis, zinc supplements can be used for the nutritional therapy for autistic patients.

## Introduction

Both Autism Spectrum Disorder (ASD) and autism are general words referring to a range of complicated brain developmental disorders. They are commonly categorized based on the differences in their degrees, which include difficulties in performing social interactions, communications-whether verbal or non-verbal, and frequent behaviors (1).

This group of disorders includes autistic disorder, Asperger syndrome (AS), childhood disintegrative disorder (CDD), and pervasive developmental delay not otherwise specified (PDD-NOS). ASD is a scarce phenomenon; however, the prevalence rate for this disorder is around 20/10,000 births (2). Both genetic and environmental factors are important in etiology of autism; of course genetic determinant is still elucidated. Toxic elements such as lead, mercury and deficiency of nutrients as well as trace elements are known as environmental factors. In comparison with healthy individuals, autistic patients, have different levels of trace elements like copper, magnesium, and zinc (3, 4). Trace elements have been proven to influence the brain neurotransmitter metabolism significantly.

Since a diagnostic test, for example, brain scan or blood test for the early diagnosis of autism does not exist; measurement of these elements can be used as a diagnostic parameter (5, 6).

One important elements of cell signaling is zinc, which plays a vital role in enzyme function, nucleic acid metabolism, growth, and finally cellular repair, most importantly in pregnant women and newborns. Zinc ions conduct an essential role in active site of more than 300 kinds of enzymes, and zinc-finger sequences exist in about 10% of the total gene-coded proteins. 

Zinc deficiency might be a major factor in the etiology of behavioral and mood disturbances in humans. Zn deficiency is high in children diagnosed with ASD. 

Upon measuring zinc levels in the plasma, hair, and nails of autistic patients, concentrations of this trace element were not normal (7). The results of the investigations are not in agreement, as in some cases individuals with ASD have had zinc deficiency, while the plasma zinc concentrations in autistic patients were not reported differently from neurotypical children (2, 8, 9). The concentrations of zinc and CU/Zn in plasma, as well as those of the hair and teeth have a relationship with the severity of the symptoms in autistic patients (10, 11, 12, 13). Since there is not yet a definite treatment for this disease therefore, medical nutrition therapy, and use of dietary supplements containing zinc can be a good solution.

Due to the lack of similar results as well as considering the impact of this element in symptoms and improve of this disease and in order to authenticate studies, performing a meta-analysis seems to be necessary.

## Materials & Methods

**Study Selection**

PRISMA guidelines were all observed (14). The articles and theses published in both national and international journals were used as the source for collecting the results. In order to collect data, we looked up Google scholar, PubMed, ISI Web of Science, and Scopus for relevant medical literature from 1978 to 2014. Literature searches were applied using the keywords ‘Autism Spectrum Disorders’, ‘autism’, ‘zinc concentration’, ‘trace element concentrations’ and their combination. Qualified studies included epidemiologic research reports measuring the correlation between zinc and autism by measuring zinc concentrations in any of the following biological sample specimens: blood/serum, hair, teeth, and toenails. All the articles, which had the keywords in either their title or abstract, were included in the initial list, and other irrelevant articles were crossed out. Studies were excluded in case they were not written in English, did not offer enough data, were reviews, or could not be categorized as epidemiologic studies. 

**Fig 1:**
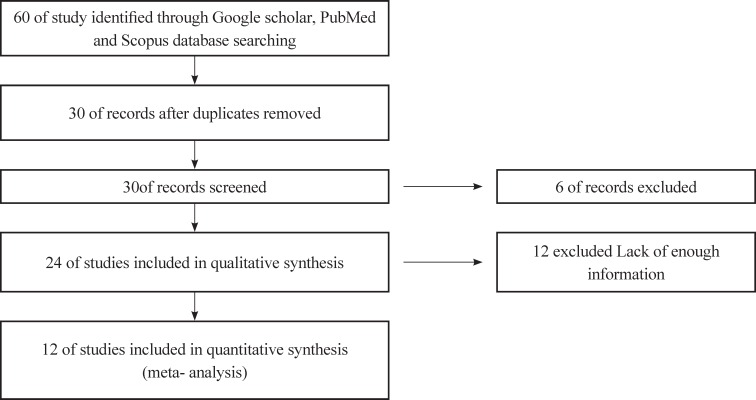
Study flowchart

The most important biases in meta-analysis are publication bias and selection bias. Publication bias was checked using Eger test. In order for the current research to minimize the probability of selection bias, the criteria were precisely defined and investigated; for each study, data were collected using two researchers separately, and the final list was prepared by consensus. Then, an information checklist for research papers consisting of sample characteristics (first author’s last name, year of publication, sample size, sample age, location), zinc concentrations, Mean difference, zinc screening method and sample specimens (Figure 1-study flowchart). 

**Statistical Analysis**

In order to merge the studies, sample size, mean, and standard derivation were considered as the criteria. Due to the few studies incorporated, and as these few studies had been already published in reputable journals, there was no room for the application of the quality criteria in the analysis. The difference between the average variance of the normal distribution was calculated using the formula of two integrated variance. The mean difference was calculated through the formula $d=\frac{{\overset{\text{̅}}{x}}_{1-}{\overset{\text{̅}}{x}}_{2}}{s}$in which ${\overset{\text{̅}}{x}}_{1}$ is the case mean, ${\overset{\text{̅}}{x}}_{2}$ is the control mean, S is the pooled sample standard deviation defined as $s=\sqrt[{2}]{s^{2}}$ which is calculated using the were $s^{2}=\frac{\left(n_{1}-1\right)s_{1}^{2}+\left(n_{2}-1\right)s_{2}^{2}}{n_{1}+n_{2}}$ the variances of the case group and control group, respectively, n1 and n2 equals the number of participants in each group. To assess heterogeneity of the studies, Cochran test and the I2 index were used.

Due to significant heterogeneity in the studies, a model with random effects was used. In order to examine publication bias Begg Plot and regressions method were used. P-value less than 5% was considered as a significant heterogeneity test. Sensitivity analysis were specified already. Statistical analyses were conducted using STATA version 12 (15, 16).

## Results

The initial search returned 60 citations. Out of which, 29 studies were discarded after reviewing the abstracts, and the remaining 21 citations were fully examined with their complete texts in more detail, 12 were appropriate for inclusion in the meta-analysis. The standard unit for measuring zinc concentrations in many articles was microgram per gram (μg/gr). However, all studies were analyzed in terms of micrograms per gram. Summary characteristics of the 12 studies are presented in Table 1. 

**Table 1:** Study Characteristics

First author (Year Publish)	Country City	Mean age		Case	Control	Matrix	Zinc concentration± SD		95% CI		Mean difference	type of Zinc measurement
		Case	Control									
							Case	Control	Lower	upper		
JacksonM.J(1986)^16^	London	7-16	7-17	20	30	plasma	0.1354± 0.0184 µmol/l	0.145± 0.015 µmol/1	-1.162	-0.007	-0.584	Atomic Absorption Spectroscopy
Shearer,T. R (1982)^24^	USA	8.4 ± 0.6	8 ± 0.8	12	12	Hair	175± 73 ppm	158 ±57 ppm	-0.734	0.867	0.067	Atomic Absorption Spectroscopy
Wecker,L(1985)^25^	USA	2-11	2-11	12	21	Hair	128±16 ppm	166±12 ppm	-1.408	0.050	-0.679	Atomic Absorption Spectroscopy
Adam.J.B(2006)^2^	USA	3-6	3-6	51	40	Hair	147±54 µg/g	134±41 µg/g	-0.149	0.683	0.267	Inductively Coupled Plasma–Mass spectrometry (ICP-MS)
Adam, J. B(2007)^3^	USA	6.1 ± 2.2	7 ± 1.7	16	11	teeth	100 ± 20 µg/g	98 ± 16 µg/g	-0.660	0.876	0.108	flame atomic absorption spectrophotometer
Priya, M. D. L(2010)^19^	India	4-12	4-12	45	50	Hair	157.66±19 μg/g	171.68±20.6 μg/g	-1.229	-0.390	-0.810	Atomic Absorption Spectroscopy
Priya, M. D. L(2010)^19^	India	4-12	4-12	45	50	Nail	176.76±21.2 μg/g	193.98±23.27 μg/g	-1.189	-0.354	-0.772	Atomic Absorption Spectroscopy
Russo.A.J(2011)^21^	USA	38	42	73	16	Plasma	78.36±20.32 mg/dl	84.42±24.18 mg/dl	-0.831	0.254	-0.288	ICP-MS
Russo, A, J(2011)^23^	USA	11.7 ± 5.62	11.7 ± 5.62	79	18	Plasma	78.36±20.32 mg/dL	84.42±24.18 mg/dL	-0.801	0.226	-0.288	ICP-MS
Elsheshtawy.E(2011)^14^	Egypt	4.1 ± 0.8	4.1 ± 0.8	32	32	Hair	304.99±25.8 mg/mg	419.5± 45.96 mg/mg	-3.802	-2.343	-0.073	Atomic Absorption Spectrophotometer
Busch, E. B(2011)^8^	Egypt	5.29±1.9	6.25 ± 2.39	25	25	Hair	101.042±52.0 mg/kg	149.86±58.51 mg/kg	-1.464	-0.300	-0.882	ICP-MS
Adam, J. B(2011)^1^	USA	5.16	5.16	55	44	Plasma	551±68 μg/dL	555±74 μg/dL	-0.543	0.340	-0.057	ICP-MS
AL-Farsi.Y.M(2012)^6^	Oman	3_14	3_14	27	27	Hair	5.4 ±0.82 μg/g	2.9 ±2.2 μg/g	0.899	2.113	1.506	ICP-MS

Seven studies were conducted in the United States, 1 was from Europe, 2 were from Asia and 2 were from Africa. 

There were three sample sources: 1: case counting from hospital records in the national health system, five studies (12, 17, 18, 19, 20); 2:Selection from special school, three studies (21, 22, 23) and one sample was from local primary schools (13); and 3: random selection from the general population, three studies (10, 24). 

The included studies involved 773 participants. In six studies, zinc status was based on analysis of hair, in 4 studies plasma sample specimen was used. Moreover, one study teeth zinc status was used. In one study in addition to hair zinc level, nail specimen also was examined (22).

Seven studies showed a significant difference of zinc status between controls and individuals with autism whereas in the remaining five, there were no significant difference of zinc statuses controls and patients. Zinc concentrations were measured in plasma, hair, nail and teeth. In this study due to non-uniform methods of zinc concentration measurement, levels of Zn in various subgroups in both cases and controls were measured.

There was not significant statistical difference between hair, nail, and teeth zinc statuses between controls and autistic patient; mean difference: -0.471 [95% confidence interval (95% CI): -1.172 to 0.231]. There was significant statistical difference between plasma zinc concentration between autistic patients and healthy controls; mean difference: -0.253 (95% CI: 0.498 to -0.007) and using a random effects model, the incorporation of data from the two groups revealed no significant meaningful difference between Zinc status in general. Mean difference: -0.414 (95% CI: -0.878 to -0.051).

**Fig 2:**
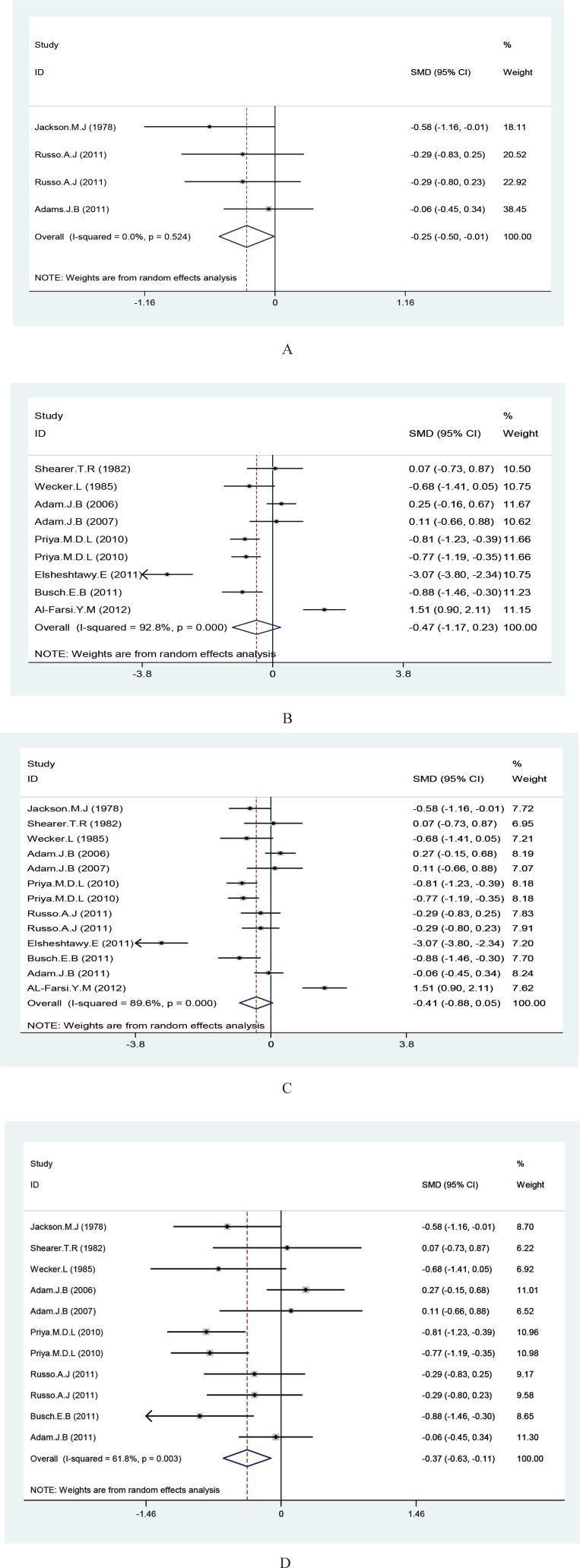
Forest plots for the Zn statues difference between autistic patients and healthy individuals. A) Plasma Zn statues, B) hair Zn statuses, C) studies Zn statuses combination, D) sensitivity analyses. The area of each square is proportional to the percentage weight of each individual study in the meta-analysis (CI 95%). In this chart studies are stored in order of year publication and author’s names, based on a random effects model

**Fig 3:**
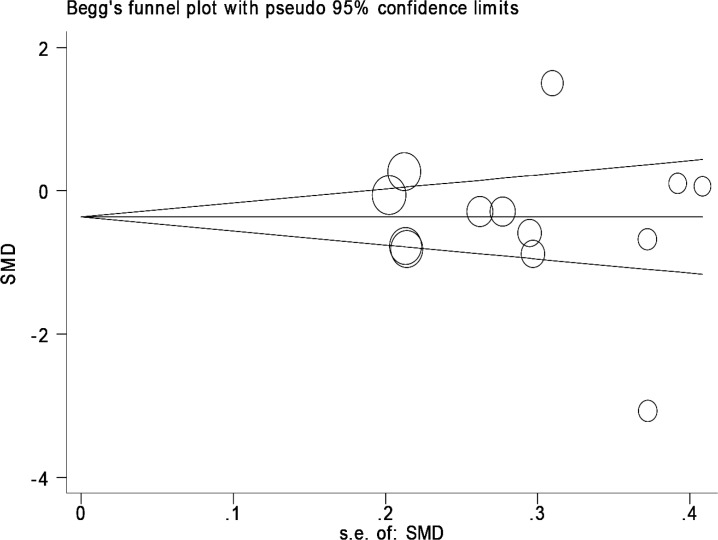
Beggs funnel plot for publication bias

Figure 2 shows the results of meta-analysis for each study and studies combination based on random effects model. Additionally, sensitivity analyses by running meta-analysis using just the higher quality studies were conducted and by excluding the 19 and 26. There were associations found (Figure 2, D). These charts are given based on years of research and the author’s name publication bias was detected by drawing Beggs funnel plot in the meta-analysis. This diagram shows there is no significant publication bias (P=0.663); this implies that each of the tests (with positive and negative results) had already been published (Figure 3).

## Discussion

In this meta-analysis, the plasma and hair status of zinc were examined. Analysis of plasma zinc concentrations has proven there was a significant difference of zinc levels between two groups of patients and controls (P=0.044). Average Zinc level in healthy subjects have been higher than that of the autistic individuals. 

Whereas the results were obtained from the hair, one nail and one teeth sample showed no significant difference in zinc concentrations between autistics and healthy subjects (P=0.189). The results of this meta-analysis were showed in overall twelve studies analysis, which applied individual levels of zinc measured in plasma and hair. There was no significant difference in zinc levels between autistic patients and healthy individuals (P=0.000). Sensitivity analyses were conducted and by excluding the 19 and 26 the associations were found. 

Individually, some studies, included in this review, had shown a significant relationship between Zn deficiency and autism (11, 19, 22). Other studies have not supported such findings (24, 25, 26). Some studies have shown the lower levels of zinc in autistic patients although their results were not significant (10, 13, 20, 21, 23, 27). 

Autism is a devastating condition with no known cure. Medical nutrition therapy accompanied by dietary supplements can be proposed as a suggestion to cure the disease (28). However, it has not been recommended to use zinc supplements as an autism treatment. Zinc concentrations in autistic children were higher than controls (24). The results from another study showed higher zinc level in autistic patients and this event could be traced to ASD (26).

Many risk factors are included in etiology of autism; one is essential elements deficiency. Therefore, it is important to determine trace element concentrations in human bodies in order to observe and investigate their effect on health (27, 29). Zinc is an essential trace element, which is of great importance in the synthesis of nucleic acid/protein, replication of cells, and growth and repair of tissues. There are some reasons showing the role of GABA in autism etiology (30). GABA levels as a neurotransmitter is reduced in autistic individuals. This amino acid is reduced leading to restlessness, aggressiveness, irritability, and seizures. 

Zinc is associated with GABA and glutamate regulation, especially through anxiolytic activity, anxiolytic activity, modulating GABAergic inhibition and seizure susceptibility (12, 26, 31). There is no evidence to support Zn supplementation in the treatment of ASD or for improving behavior and cognition in ASD giving the important role of Zn in neuronal function (32). The supportive laboratory data, and the fact that children with ASD might already have a deficiency and according to sensitivity analysis, which have been performed in the current study, Zn supplementation may be an important novel treatment to investigate in the future (20, 27, 33). 

Nevertheless, the results of this meta-analysis zinc deficiency might not be a major factor in the etiology of behavioral and mood disturbances in humans. 

The major limitation is the conduct of a meta-analysis in the presence of high heterogeneity among the studies. In order to relieve the heterogeneity, random effects model was applied and the results were changed according to the sensitivity analysis.

Methodology applied to the studies, which were reviewed here, is commonly considered as a limitation and weakness of the current study. Among the mentioned weaknesses the following can be presented as an instance: 

1) Lack of a same method of measurement for the variances.

2) Lack of information about nutrition and lifestyle of participants.

3) Various kinds of screening methods and lack of same standard unit for measuring zinc concentrations in different articles.

4) One significant limitation was lack of access to some relevant studies.

**In conclusion, **The current meta-analysis, conducted regarding random effect model, revealed that generally, there is not any significant relationship between Zinc levels and autism. Nevertheless, a difference was observed between the plasma Zn levels of ASD patients and that of the controls. Moreover, taking sensitivity analysis into account and upon excluding 19 and 26, it can be concluded that zinc supplements can be used in clinical trials and randomize clinical trials for the nutritional therapy for autistic patients.

## References

[1] Arnold LE, Farmer C, Kraemeret HC (2012). Moderators, mediators, and other predictors of Risperidoneresponse in children with Autistic Disorder and Irritability. J Child Adolesc Psychopharmacol.

[2] Karimzadeh P (2000). Recent finding about etiology of autism. Rehabilitation.

[3] Dufault R, Schnoll R, Lukiw WJ (2009). Mercury exposure, nutritional deficiencies and metabolicdisruptions may affect learning in children. Behav Brain Funct.

[4] Morris CR, Agin CM (2009). Syndrome of allergy, apraxia, and malabsorption: characterization of a neurodevelopmental phenotype that responds to omega 3 and vitamin E supplementation. Altern Ther Health Med.

[5] An centers for Disease Control and Prevention (2012). Prevalence of autism spectrum disorders-Autism and Developmental Disabilities Monitoring Network, Prevalence of Autism Spectrum Disorders (ASDs) Among Multiple Areas of the United States in 2008, United States, Morbidity and Mortal Weekly Report (MMWR).

[6] Dufault R, Lukiw WJ, Crider R (2012). A macro epigenetic approach to identify factors responsible for the autism epidemic in the United States. Clin Epigenetics.

[7] Faber S, Zinn GM, Kern GC (2009). The plasma zinc/ serum copper ratio as a biomarker in children with autism spectrum disorders. Biomarkers.

[8] Cornish E (2012). Gluten and casein free diets in autism: a study of the effects on food choice and nutrition. J Hum Nutr Dietet.

[9] De Palma G, Catalani S, Franco A (2012). Lack of correlation between metallic elements analyzed in hairby ICP-MS and Autism. J Autism Dev Disord.

[10] Adams JB, Romdalvik J, Ramanujam VM, Legator MS (2007). Mercury, Lead, and Zinc in Baby teeth of children with Autism versus controls. J Toxicol Environ Health A.

[11] Blaurock-Busch E, Amin OR, Rabah T (2011). Heavy metals and Trace elements in hair and urine of a sample of Arab children with Autistic Spectrum Disorder. Maedica (Buchar).

[12] Russo AJ, Devito R (2011). Analysis of Copper and Zinc Plasma Concentration and the efficacy of Zinc therapy in individuals with Asperger’s syndrome, pervasive Developmental Disorder Not Otherwise Specified (PDDNOS) and Autism. Biomarker Insights.

[13] Shearer TR, Larson K, Neuschwander J, Gedney B (1982). Minerals in the hair and nutrient intake of Autistic children. J Autism Dev Disord.

[14] Liberati A, Tetzlaff J, Mulrow C (2009). The PRISMA statement for reporting systematic reviews and metaanalyses of studies that evaluate healthcare interventions: explanation and elaboration. BMJ.

[15] Hartung J, Knapp G, Sinha BK (2008). Statistical Meta- analysis with application.

[16] Babaknejad N, Sayehmiri F, Sayehmiri K (2014). The relationship between selenium levels and breast cancer: a systematic review and meta-analysis. Biol Trace Elem Res.

[17] Al-Ayadehi LY (2005). Heavy metals and trace elements in hair samples of autistic children in central Saudi Arabia. Neurosciences (Riyadh).

[18] Blaurock-Busch E, Amin OR, Dessoki HH, Rabah T (2012). Toxic metals and essential elements in hair and severity of symptoms among children with Autism. Mædica J Clin Med.

[19] Elsheshtawy E, Tobar S, Sherra K (2011). Study of some biomarkers in hair of children with autism. MECPsych.

[20] Russo AJ (2011). Increased Copper in individuals with Autism normalizes post Zinc therapy more efficiently in Individuals within current GI Disease. Nutr Metab Insights.

[21] Jackson MJ, Garrod PJ (1978). Plasma Zinc, Copper, and Amino Acid levelsin the blood of Autistic Children. J Autism Child Schizophr.

[22] Priya MDL, Geetha A (2011). Level of trace elements (Copper, Zinc, Magnesiumand Selenium) and toxic elements (Lead and Mercury)in the Hair and Nail of Children with Autism. Biol Trace Elem Res.

[23] Wecker L, Miller SB, Cochran SR, Dugger DL, Johnson WD (1985). Trace element concentrations in hair from autistic children. J Ment Defic Res.

[24] Adams JB, Audhya T, McDonough-Means S (2011). Nutritional and metabolic status of children with autism vs neurotypical children, and the association with autism severity. Nutr Metab (Lond).

[25] Adams JB, Holloway CE, George F, Quig D (2006). Analyses of toxic metals and essential minerals in the hair of Arizona Children with Autism and associated conditions, and their mothers. Biol Trace Elem Res.

[26] Al-Farsi YM, Waly MI, Al-Sharbati MM (2013). Levels of heavy metals and essential minerals in hair samples of children with Autism in Oman: a Case–Control Study. Biol Trace Elem Res.

[27] Russo AJ (2009). Decreased serum Cu/Zn SOD in children with Autism. Nutr Metab Insights.

[28] Xia W, Zhou Y, Sun C, Wang J, Wu L (2010). A preliminary study on nutritional status and intake in Chinese children with autism. Eur J Pediatr.

[29] Russo AJ, Bazin AP, Bigega R (2012). Plasma Copper and Zinc Concentration in Individuals with Autism Correlate with Selected Symptom Severity. Nutr Metab Insights.

[30] Bjørklund G (2013). The role of zinc and copper in autism spectrum disorders. Acta Neurobiol Exp.

[31] Yasuda H, Yoshida K, Yasuda Y, Tsutsui T (2011). Infantile zinc deficiency: Association with autism spectrum disorders. Sci Rep.

[32] Frye RE, Rossignol D2, Casanova MF (2013). A review of traditional and novel treatments for seizures in autism spectrum disorder: findings from a systematic review and expert panel. Front Public Health.

[33] Yasuda H, Tsutsui T (2013). Assessment of Infantile Mineral Imbalances in Autism Spectrum Disorders (ASDs). Int J Environ Res Public Health.

